# A mechatronic shirt kit to enhance psychomotor and life skills in autistic children: a pilot study

**DOI:** 10.1038/s41598-025-20597-3

**Published:** 2025-10-21

**Authors:** Ramya S Moorthy, R. Hari Krishnan, S. Pugazhenthi

**Affiliations:** 1 Department of Mechatronics, Manipal Institute of Technology, Manipal Academy of Higher Education, Manipal, Karnataka 576104 India; 2 School of Mechanical Engineering, SASTRA Deemed to be University, Thanjavur, Tamil Nadu 613401 India

**Keywords:** Autism training, Daily life skills, Elbow movement, Hand-eye coordination, Mechatronic-shirt kit, Pincer grasps, Psychomotor skills, Social robotics, Health care, Engineering, Biomedical engineering, Electrical and electronic engineering, Mechanical engineering

## Abstract

Training autistic children has become a global challenge, as the number of children diagnosed with it is increasing drastically. The conventional therapies to train autistic children have their own limitations. Technology-enabled training kits can be developed to help impart psychomotor skills to these children. Studies have reported that robot or robot-like features attract autistic children. Taking this as a leverage, in this work, a mechatronic kit has been developed to impart psychomotor and cognitive skills that are useful in day-to-day activities, especially when wearing a shirt. The psychomotor skills addressed are pincer grasp, elbow movement and hand-eye coordination, while the cognitive skills addressed are identification of shirt, colour identification and matching. Pilot trials have been conducted with seven autistic children to study how useful the kit is in imparting the targeted skills. The trials consisted of pre-assessment, training, and post-assessment sessions, having 15 tasks spanning over 7 sessions and 68 trials. After the training, from pre-assessment to post-assessment, an improvement of around 80% has been observed in the Daily Life Skills (DLS) of connecting the shirt with velcros and its associated psychomotor skills.

## Introduction

The United States Centers for Disease Control and Prevention (CDC) has disclosed that, in America, 1 in 36 children under the age of 8 years come within the sphere of Autism Spectrum Disorder (ASD)^1^. Throughout the world, the prevalence of autistic children is on the rise. Autism occurs in infancy and persists throughout life, with impairments in communication and socialisation, which questions the ability to meet the demands of the day-to-day life of an individual^2^. Apart from deficits in communication and socialisation, more than 80% of the autistic children have gross and fine motor skill deficits, lack of postural control and praxis impairments^3,4^. Various reports have indicated that these children have motor skill deficits significant enough to meet the DSM-IV (Diagnostic and Statistical Manual of Mental Disorders) criteria^5,6^. As latest as 2023, rehabilitative trials conducted with 5 to 6-year-olds of both neurotypical children and with ASD have proved that autistic children have severe psychomotor skills deficits^7^. In a very recent study involving 196 children with various Neuro Developmental Disorders (NDD) like ASD, language disorder and Mixed Specific Developmental Disorder (MSDD), autistic children exhibited poor psychomotor skills^8^. Another recent study involving 108 children reported that around 80% of these children displayed motor challenges or were at risk of developing such issues in the near future^9^. Farazao et al.^10^ have consolidated and reported various intervention practices currently followed for preschool children on the spectrum, but they doubt the effectiveness of these conventional practices. Through the intervention for Fundamental Motor Skills (FMS), improvements in both motor skills and individual behaviour were observed, which had been left unobserved by previous researchers in this field^11^. The psychomotor deficits in autistic children result in disruption of Daily Life Skills (DLS) like eating routines, washing, dressing in a consistent way, and so on^12,13^. Thus, learning DLS is essential to function independently and successfully^14^ and also contributes to the prognosis of the child^15^. The sensorimotor skills are highly related to DLS, and interventions to support and address these skills are of high priority^13^. A study that assessed 35 preschool children on the spectrum reported that the children who habitually avoided sensory input and those who were weak with fine motor abilities exhibited significantly lower DLS^16^. This substantiates that the sensorimotor development is foundational for daily living routines. Developing interventions for autistic children targeting DLS deficits have been of interest for some time, as ascertained by Kanner^17^.

The common therapies used until today for interventions are occupational therapy, speech therapy, and physiotherapy^18^. However, other alternative and exploratory therapies are being used in adjunct to conventional therapy systems to increase benefits^19^. Among modern therapies, cognitive remediation, intending to improve cognitive processes such as attention, memory, social cognition and so on, is also gaining momentum^20^. Though they are effective to some extent, there are some drawbacks. One major drawback is the challenges in child-trainer communication. During training, autistic children always find it difficult to communicate with the trainers as they lack communication skills and are unwilling to make eye contact^21,22^. The robotic kits, on the other hand, make the children feel more comfortable, breaking the barrier for communication. The children show an increased level of motivation and engagement with tasks that they are subjected to, using robots, as compared to similar tasks without the robots^23^. Begum et al. suggest that autistic children exhibit interest towards robots and robot-like features and also have an extraordinary bond with them than with humans^24^. They also highlight the importance of interventions involving robots over conventional therapy methods. Also, robotic intervention can incorporate elements of gamification which has demonstrated an increase in attention of autistic children during training^25^.

As a part of research in embodied interaction, a number of robots have been developed. Nao, Keepon, CHARLIE, Zeno, KASPAR, PARO, iRobiQ, CARO, Romibo, ROMO, DARWIN-MINI, and so on are a few of the social robots used in rehabilitative therapies to enhance skills like imitation, social interactions, adaptation to changes and turn passing^21,26–30^. Therapists use Nao robot as a tool to deliver social stories to autistic children, making it easier for the children to understand^31^. Holeva et al. have reported that using the same Nao robot for intervention with autistic children shows an increase in gesture actualisation, speech and most importantly, eye contact by the end of the training^32^. In another study Nao robot was employed in a pre-school setting to examine the relationship between children—teachers, and children—Nao robot during motor activity sessions^33^. CHild-centred Adaptive Robot for Learning in an Interactive Environment (CHARLIE) engages the children through a simple imitation and turn-taking game, equipped with a face tracking mechanism and camera that observes the progress of the children^34^. In the case of Zeno, motions of both the child and Zeno are recorded and compared, using simple position control schemes, to see the progress of the child^35^. Another robot, Keepon, which is a small creature-like robot, is used for natural, simple and nonverbal interaction with children^36^. As a part of the AURORA project, a KASPAR robot is used as a social mediator that encourages autistic children to interact with the robot and thereby breaks their isolation and facilitates interactions with other people^37^. An easy-to-use, cost-effective toy robot was used to train autistic children to enhance their social skills, and the trials conducted showed a positive impact on the training outcomes^38^. Soleiman et al. had reported that creating a Robotic Social Environment (RSE) helps in enhancing recognition of basic facial expressions like anger, sadness, happiness and fear by autistic children^39^. The RSE has been created by developing two different coloured parrots with an audio output, talking about the facial expressions. JARI is a social robot developed to help autistic children improve their ability to recognise emotions and participate in social interactions^40^.

Even though researchers have focused on developing robots for training autistic children, they are predominantly for the enhancement of social interaction, facial expression, cognition, etc., and not for psychomotor skills. Focusing on enhancing psychomotor skills, a simple OWI pick and place robotic arm was customised to impart skills such as palmar grasp, hand-eye coordination and knowledge of directions (up, down, right and left)^41^. Similarly, another mechatronic training kit has been developed to address psychomotor skills, including wrist rotation, palmar grasp, and eye-hand coordination, in addition to teaching them the principle of closing and opening of doors^42^. Other than robots, a study aiming at the potential use of Virtual Reality (VR) to aid autistic children in improving gross motor skills has been reported recently^43^. These papers focus on imparting psychomotor skills to autistic children, using technological interventions, to cope with daily life activities. The mechatronic training kit developed in this work is aimed at training autistic children on daily life activities of wearing shoes and shirts. The kit targets the enhancement of relevant psychomotor skills like pincer grasp, elbow movement, hand-eye coordination and other cognitive skills that are required to perform these tasks. To assess the effectiveness of the kit, pilot trials have been carried out involving autistic children, consisting of pre-assessment, training and post-assessment sessions. The results of the trials have been analysed and the key findings have been presented in this study.

## Training kit

### Skills being addressed

Imparting DLS to autistic children is an important training activity. In day-to-day life, every child needs to be adept at performing easy tasks like correctly identifying and wearing a shirt and/or shoes properly. These effortless tasks, which come easily to neurotypical children, are difficult to carry out for most of the children on the spectrum. The skills required to perform these tasks need to be taught to these children in a simple and effective manner. The psychomotor skills that are involved in these tasks are pincer grasp (fine motor), elbow movement (gross motor) and hand-eye coordination. Apart from the above psychomotor skills, cognitive skills like identification and matching are also needed. Another deficit found in autistic children is the lack of generalisation of the above skills in daily life, which also needs to be addressed through training.

### Training methodology

In order to simplify the task of learning targeted specific tasks involved in wearing a shirt or shoes, it is proposed to design a mechatronic kit, having a board onto which a shirt and a pair of shoes are fixed. The children need to be trained in the identified tasks in a step-by-step process. Autistic children have minimal articulation and dexterity of the fingers^44^ and thus, it will be difficult to teach them to button and unbutton a shirt. Hence, instead of using shoelaces and shirt buttons, Velcro fastenings are selected for training. In the process of learning, they also need to be given positive reinforcement during different stages of learning, leveraging mechatronics technology. Various researchers have reported the importance of positive reinforcement in training autistic children^45–49^. For instance, Greczek et al.^46^ reported the technique of graded cueing for health-behaviour coaching in which an imitation game is played between a Nao robot and an autistic child. In the game, the robot assumes a particular pose and verbally prompts the child to imitate the same. If the child successfully imitates the same, the robot provides a positive reinforcement in the form of verbal feedback, nods, and flashes its eyes green. These works highlight that with proper positive reinforcement, children tend to be highly engaged with the activity as they find rewards encouraging, and this positively impacts their learning.

After being trained using the kit, the children are expected to understand the concept of the shirt, connecting the velcros (once the shirt is on them by the caretaker), eye-hand coordination, pincer grasp and matching the colours. Following the use of this device, the future plan will be to focus on aiding the child to understand how to pick up the shirt, orient it correctly on their body, and insert their hands into the appropriate sleeves.

### Description and working of the system

**Fig. 1:**
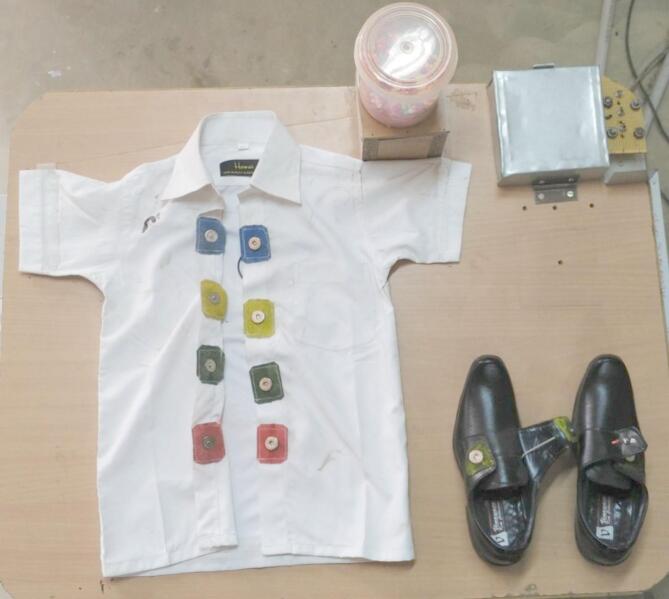
Mechatronic shirt – shoe kit.

Figure 1 shows a photograph of the developed mechatronic shirt-shoe kit. A white coloured shirt and a pair of black shoes are firmly attached to a wooden plank. The white shirt has four pairs of different coloured (blue, yellow, green and red) Velcros that are stitched instead of buttons. Similarly, the shoes have one pair of Velcros on each shoe. A canister containing small thermocol balls of various colours is placed on a raised wooden platform on the right-top portion of the plank. The shirt, shoes and the canister are placed within the zone of sight of the children. The thermocol balls will rise and rotate within the canister when the connections are correct. This will give a positive reinforcement to the child during learning. The kit employs an Arduino UNO module to control various activities involved in the kit.

The description and the training using the shoe module have been elaborately discussed in Moorthy et al.^47^. The training using the shoe module had been carried out prior to the shirt module as the shoe module is less complex than the shirt module. The skill set addressed in the shoe module were identification of the shoe, pincer grasp and differentiating between right and left shoes.

In the shirt module, onto each of the Velcro connectors within the shirt (coloured blue, yellow, green and red), magnetic metal snaps are attached to maintain reliable and robust electrical connections when joining the two sides. The male and female halves of the magnetic snap are sewn onto the hook and loop sections of the Velcro closures respectively. Out of the four Velcro closures, the odd-numbered female halves are connected to a 5 V power source and the even-numbered female halves are connected to a 3.3 V power source. This is to avoid the mismatch of the snaps. The male halves of the odd-numbered snaps are connected to analog pins A1 and A3 of the Arduino module, and the even-numbered counterparts are connected to analog pins A2 and A3. During the task, when the loop connects to the hook, the male half snaps into the female half, allowing the corresponding voltage level to be detected in the respective analog input pins. The system is programmed in such a way that the corresponding voltage level is detected when the colours are matched correctly. This triggers a DC motor, controlled by a motor driver connected to pins 8 and 12 of the Arduino UNO module, which then rotates the fan, causing the thermocol balls inside the canister to move to provide positive reinforcement. When the closures are separated from each other, the actuation of the motor is seized, which in turn stops the rotation of balls.

## Trials

Having developed the mechatronic training kit, the trials were carried out involving seven children. All children included in the study had a prior clinical diagnosis of Autism Spectrum Disorder (ASD) confirmed by a certified psychologist prior to joining the school. One child had co-occurring features of Mental Retardation (MR) in addition to ASD; no other comorbidities were present in any participant. Of the seven children, three spoke few or no words and four were semi-verbal. At the time of the trial, all participants were undergoing both speech therapy and occupational therapy in a school for children with disabilities in Tiruchirapalli, Tamil Nadu, India. No other therapeutic interventions were being administered concurrently. These have been listed in Table 1, and the authors affirm that the legal guardians of the children have provided informed consent for publication of this information. Additional personal or sensitive data have been withheld to ensure participant anonymity and ethical compliance. The training was carried out on a one-on-one basis by their respective special educators in an enclosed space arrangement so as to avoid distractions, in accordance with relevant guidelines and regulations. The study protocol was approved by the Institutional Ethics Committee (IEC) of Thanjavur Medical College, Tamil Nadu (Approval Number:534) on 1st July 2018 and trials were carried out subsequently. All the sessions were conducted and videotaped with the informed consent of the parents for further analysis. The trials were divided into pre-assessment, training and post-assessment sessions. The pre-assessment and the post-assessment sessions were carried out to observe and understand the extent of knowledge of skills of the children before the training and enhancement of the same skills after training, respectively. These trials were planned keeping social interaction as a function of reinforcement as put forward by Morris and Vollmer^50^.

**Table 1:** Demographic details of the pilot study participants.

Child	Age	Gender	Diagnosis	Communication	Additional training
1	7	Female	ASD	Few or no words	Speech and occupational therapy
2	13	Male	ASD	Semi verbal	Speech and occupational therapy
3	8	Male	ASD	Few or no words	Speech and occupational therapy
4	8	Male	ASD	Semi verbal	Speech and occupational therapy
5	10	Female	ASD	Semi verbal	Speech and occupational therapy
6	9	Male	ASD	Semi verbal	Speech and occupational therapy
7	10	Female	ASD + MR	Few or no words	Speech and occupational therapy

As this was a pilot study aimed at evaluating the potential of a novel kit and its intervention, a formal sample size calculation was not performed prior to this study. Even though the sample size is limited, the outcomes of this study provide initial insights into the effectiveness of the developed kit in interventions. Future studies with larger cohorts and proper sample size calculations are planned to further validate the findings.

### Pre-assessment session

Four distinct tasks were planned as part of the pre-assessment. A white shirt resembling the one utilised in the system is positioned before the child. The initial task was for the child to identify the shirt on command. Followed by the second task, in which the child had to connect the Velcro closures on the shirt by matching the correct colours. In the third task, four coloured balls were placed in front of the child, and he/she had to pick up the correct coloured ball and place it on the special educator’s hand when the colour was called out. The last task was for the child to connect the Velcros after the shirt was put on the child by the trainer. This task was designed specifically to see if the child was able to generalise the task of connecting the velcros despite the change in the orientation of the shirt when compared to that of the training device. The output of these activities were recorded for the purpose of comparison and analysis with the output of post-assessment activities. The shirt used in the pre-assessment and post-assessment was custom-designed with Velcro specifically for this study to enable children with disabilities to gain more independence in dressing tasks. It is also important to note that adaptive clothing featuring Velcro and snap closures (instead of buttons) is increasingly available in the market for children with disabilities^51^. These emerging clothing designs promote functional independence in dressing and support the rationale behind the approach of this paper.

### Training

The shirt training was conducted over a span of five sessions using the mechatronic training kit. While the tasks seen in the pre- and post-assessment are designed to mimic real-life scenarios, the tasks in the training modules are designed to train the child across the different chosen parameters. The children were taught to connect one colour in each of the first four sessions and connect the combination of all the colours in the fifth session of the trials. The tasks involved in the first four sessions were (1) to identify the correct colour and (2) to connect the respective connectors in the shirt. Both these tasks were carried out using any favourable hand of the child. When taught to hold one side of the Velcro connector and attach it to the other side, children learn the ‘pincer grasp,’ a fundamental grip necessary for many DLS. Every time the connection is established properly, the balls in the canister are made to vibrate, providing a positive reinforcement for the child.

#### Training procedure

In the first session, children were trained to connect the blue closures followed by yellow, green and red colours in the second, third and fourth sessions respectively. These four sessions comprised 12 trials each, dedicated for one specific colour per session. In the fifth session, five sets of combinations of all the colours were carried out, with each set comprising four trials, making it to a total of 20 trials. The total number of trials combining all the sessions results in 68 trials, the details have been tabulated in Table 2. The first three sets were carried out in the same order in which the colours were taught. In the fourth set, the colours were called in the reverse order and in the fifth set, the colours were called in a random order. This was to ensure that the children identified the colours in any order and also generalised them in everyday life. At the beginning of every new session, the previous tasks were practised before introducing a new task.

**Table 2:** Trial details.

Setting	Sessions	No. of tasks	Tasks	No. of trials
Pre-assessment	1	4	Identify shirt	1
			Connect the velcros without wearing	1
			Identify four colors	1
			Connect the velcros after wearing	1
Training—learning phase	4	4	Connect Blue velcro	12
			Connect Yellow velcro	12
			Connect Green velcro	12
			Connect Red velcro	12
Training—assessment phase	1	3	Connect the colors in same order	12
			Connect the colors in reverse order	4
			Connect colors in random order	4
Post-assessment	1	4	Identify shirt	1
			Connect the velcros without wearing	1
			Identify four colors	1
			Connect the velcros after wearing	1

**Fig. 2:**
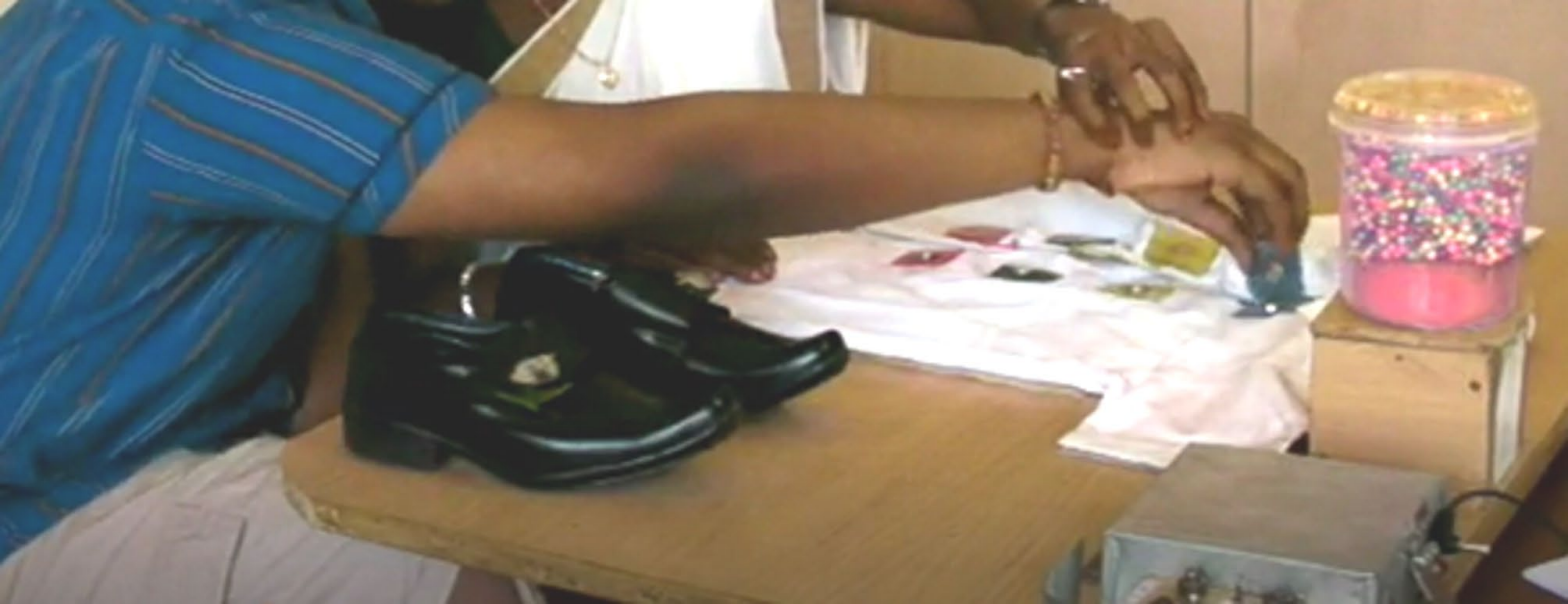
Training with shirt module.

The training was initiated by making the child sit in front of the kit with an unobstructed view of the shirt. First, the special educator demonstrated and introduced the system to the child. Then the child was made to touch the shirt by physically holding his/her hand and uttering the words “shirt” simultaneously. Subsequently, the educator held the hand of the child onto the blue Velcro closure, simultaneously uttering the word “blue colour”. Then the special educator physically held the child’s fingers into a pincer grasp and connected the blue coloured closures on the shirt. When the positive reinforcement was activated, the educator pointed to the canister and made the child look at it. After completing this training cycle for the first time, the child was then verbally instructed to connect the blue-coloured Velcro on their own. If the child did not respond, then the educator would teach it again physically. If the child responded but required assistance, then the appropriate prompts like physical cue, touch cue or oral cue, were provided to help them complete the task. In case of the need for a physical cue, the child was taught by physically holding his/her hand, whereas, in case of a touch cue, assistance was provided by just touching the child’s hand. Similarly, in case of need for an oral cue, the special educator instructed the child orally until he/she was able to carry out the task. During the second session, the second task, i.e. connecting the yellow closures, was taught in a similar manner. After every trial, a ten-second wait time was introduced so as to teach the concept of waiting, listening to commands. This helps in improving the acceptance and adaptability to a given situation. In the third and fourth sessions, connecting the green and red closures was taught respectively. Finally, on the fifth session, the children were trained to connect all the combinations as mentioned previously. Figure 2 shows an image of training a child with the shirt module of the kit.

#### Observations

**Fig. 3:**
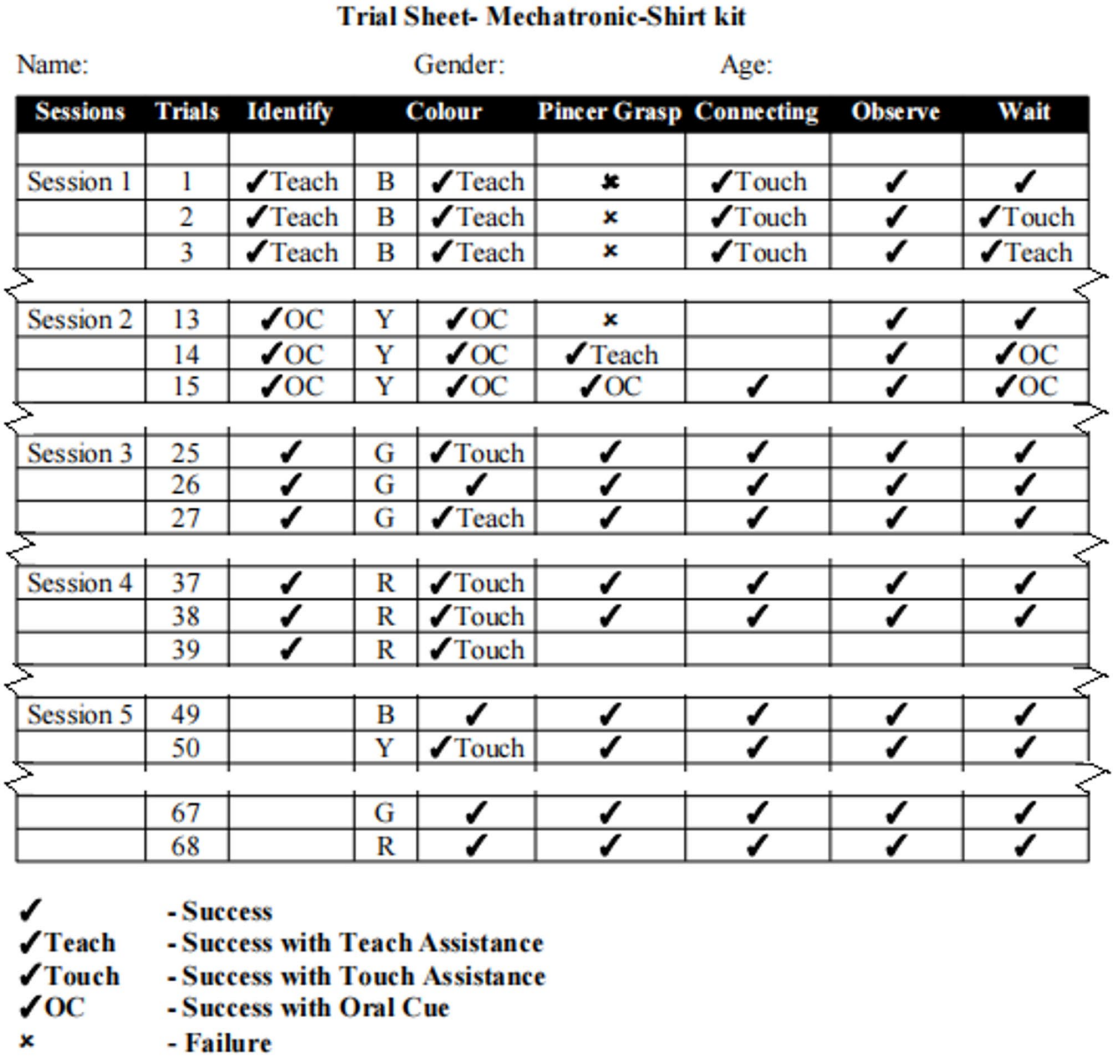
A sample trial sheet used for training with shirt module.

The outcomes of every trial of all the sessions were recorded in dedicated trial sheets similar to the one shown in Fig. 3. A trial sheet consists of different rows and columns, which correspond to trials and skills respectively. Depending on the assistance required for each of the trials, the markings were done. If the child connected the correct colour using pincer grasp ✔ was marked in the corresponding cells of ‘Colour Identification’, ‘Pincer grasp’ and ‘Connection’ column. If any sort of assistance was provided to the child, then a ✔ was marked followed by the prompt required i.e., ‘Teach’, ‘Touch’ and ‘OC’ for physical cue, touch cue and oral cue respectively. If the child did not respond, then ✘ was marked in the corresponding cell. A similar marking system was used to mark the ‘wait’ column, where the child was instructed to wait for 10 seconds before the next trial began. The ‘observe’ column was marked appropriately each time the child observed the canister after a successful trial.

For evaluation of the training and subsequent analysis, during each trial, scores were provided for various associated skills like ‘colour selection’, ‘pincer grasp’ and ‘connection’. Scores between 0 and 4 were assigned depending on the performance of the children based on the markings in the trial sheet, corresponding to different markings, as per Table 3.

**Table 3:** Assignment of scores.

Observations	Markings	Scores
Failure	·	0
Success with physical cue	✔Teach	1
Success with touch cue	✔Touch	2
Success with oral cue	✔OC	3
Success	✔	4

The overall weighted score for rating the performance of a task in a particular trial is achieved by first providing weights of 0.2, 0.3 and 0.5 for the associated skills, namely identification of the colour, hold using pincer grasp and connection of closures respectively. Subsequently, the assigned scores are multiplied by the corresponding weights to compute the weighted score for each skill. The total weighted score, which indicates the child’s performance in a specific task during each trial, is calculated by summing the weighted scores of the skills, out of a maximum possible score of four. Similarly, the performance assessment (total weighted score) of every child during all 68 trials was carried out as explained above.

The weighted scoring system has been formulated to represent the relative complexity of the relevant skills that have been assessed, namely identification of colour (0.2), pincer grasp (0.3), and connection of closures (0.5). The highest weight (0.5) has been assigned to the connection of closures as it requires psychomotor integration, involving both cognitive and motor components. In this task, the child is expected to identify the closure, plan and sequence the action, and execute it by moving the hand towards it using gross motor control and fine motor coordination. It best represents the core objective of the device. A moderate weight of 0.3 has been given to the pincer grasp as it is a fine motor skill that is essential for manipulation and control. It involves only a minimal cognitive engagement. The lowest weight (0.2) has been provided to the identification of colour as it is a purely cognitive task involving visual discrimination and no physical execution. This hierarchy of weights ensures that the scoring system prioritises tasks that challenge both cognitive and motor domains, in alignment with the intervention’s goals.

### Post–assessment

Post-assessment was conducted after the completion of the training sessions to assess the improvement in the children. The same four tasks that were conducted in the pre-assessment session were conducted in the post-assessment in the same order and the outcomes were recorded for further analysis.

## Results and discussions

The outcomes that were recorded in the trial sheets were used for evaluating the training by plotting different graphs. The graphs were split into learning and assessment graphs. The first four sessions were considered as learning curves and the five sets of trials in the fifth session were considered as assessment curves.

### Learning and assessment curves

**Fig. 4:**
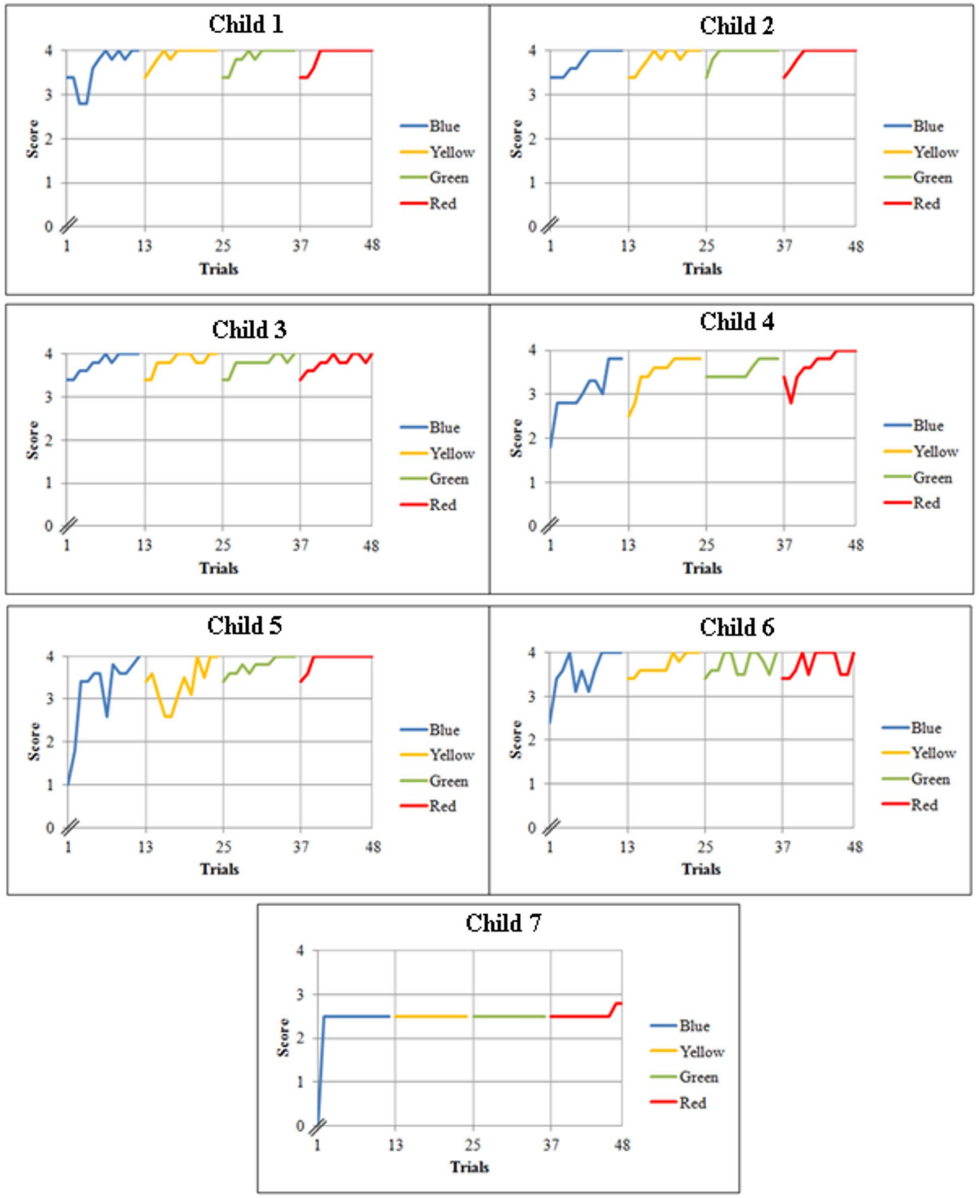
Learning curves of all the children.

Two sets of graphs were drawn using the weighted score for all seven children. The first set as shown in Fig. 4, comprises learning curves of the first four sessions and it is divided into four parts. The first 12 trials correspond to connecting the blue colour, the second 12 to yellow. Similarly, the third and the fourth correspond to connecting green and red respectively. From the figure, significant improvement can be observed not only at the end of the first four sessions but also at the end of every training session for all the children. The graph also indicates that though the learning curve is unique for every child, all the children showed improvement throughout the training.

In the case of child 1, it can be seen that the child required physical assistance initially to identify the blue colour and pincer grasp, but was able to carry it out at the end of the first session. During the next three subsequent sessions, the child required assistance at the beginning, only for identifying the colours but was able to perform pincer grasp and connection without any assistance. Later, towards the end of each training session, the child was able to achieve the top score of 4. This indicates that the child required help when a new colour was introduced, as it was a new learning, repeated practice was required for her to achieve it. A similar learning trend was found in children 2, 3 and 6. They all required assistance at the start of each session to identify the colours but were able to execute the task successfully by the end of each of the sessions. Child 5, in addition to identifying colours, required assistance initially for executing the pincer grasp and connection of the Velcros as well. Nevertheless, the child achieved a top score towards the end of the training sessions. In the case of child 4, it can be seen from the graph that, unlike the other children, the child needed minimal assistance (oral cues in this case) to identify the colours blue, yellow and green. However, towards the end of the fourth session, the child carried out the task successfully without any assistance. Child 7 demonstrated the lowest performance compared to all others, requiring assistance throughout the training sessions to complete all tasks. On the first trial the child did not respond to the given instruction at all thus scoring zero, but from the second trial onwards the child was able to perform the task with physical assistance for all the skills. Until the 46th trial the child had a constant score of 2.5 after which the child was able to carry out pincer grasp with a touch cue, scoring a 2.8. This could be associated with the fact that this child had also been diagnosed with Mental Retardation (MR) features apart from ASD. With the repeated practice of the activity, there are possibilities of improvement in the scores of this child. However, more trials with similar sets of children (MR with ASD) need to be carried out in the future to come to any concrete conclusion.

‘Observe’ and ‘wait’ were the other two skills that were considered during the training. If the children had observed the positive reinforcement produced by the movement of balls in the canister then the ‘observe’ was noted. Similarly, if the children had waited between the trials, ‘wait’ was noted. In both cases, except child 7 all the other children required assistance only at the beginning of the training but were able to obey the same commands towards the end of the training session without any assistance. However, child 7 needed sporadic minimal assistance throughout the entire training period, for reasons already discussed above.

**Fig. 5:**
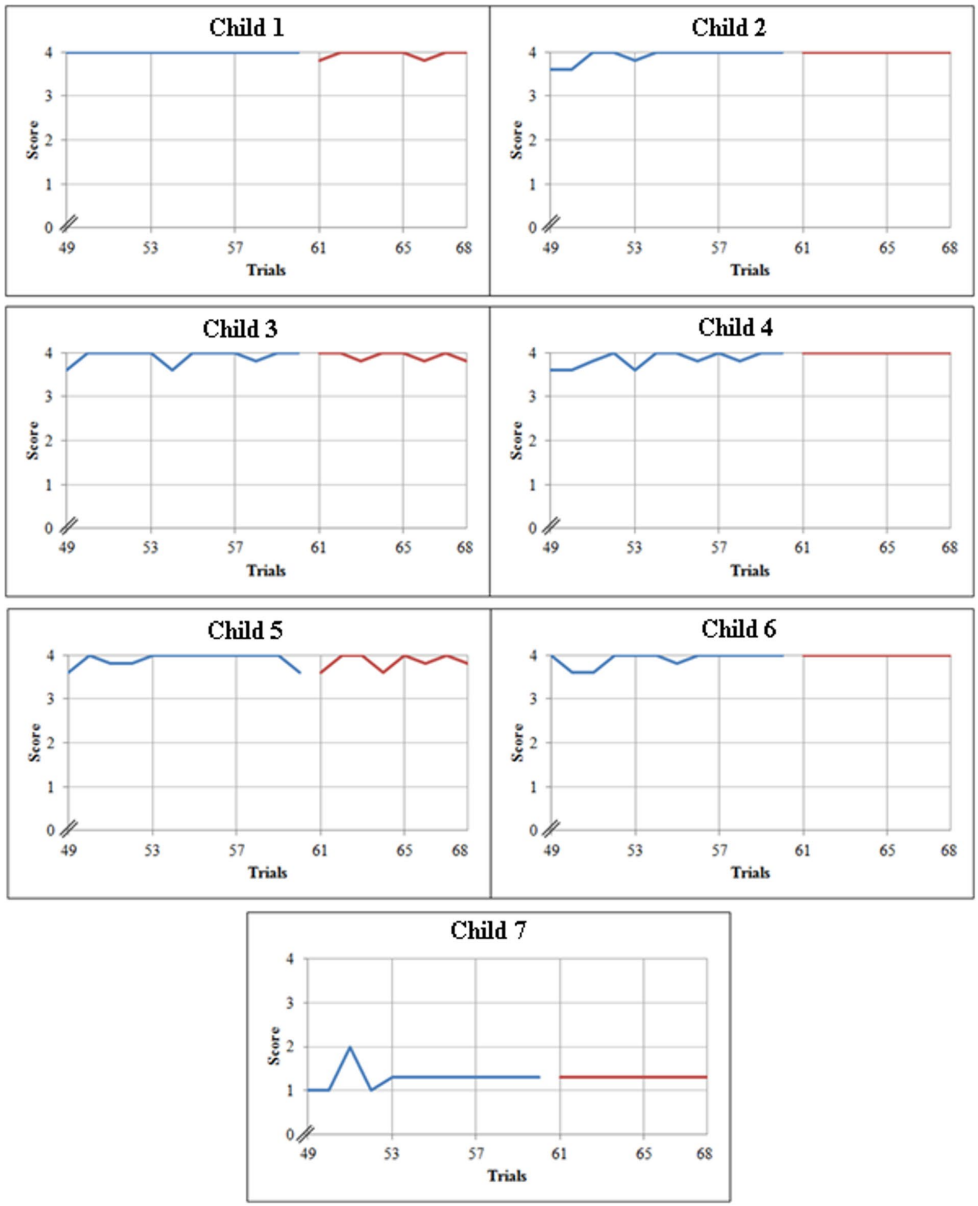
Learning and assessment curves of all the children.

After plotting the first set of graphs that comprises learning curves of first four sessions, the second set of graphs comprising assessment curves of the fifth session of all the children were plotted as shown in Fig. 5. Here the first three sets of trials that are from 49 to 60 were the outcomes of combination of all the four colours in the same order as taught in previous sessions. Trials 61–64 correspond to combinations of all four colours in reverse order and trials 65–68 correspond to colours in random order.

It can be seen from the graph that the performance at the start of the fifth session was consistent with the performance at the end of the previous session, as most of the children were able to perform the task without any or with minimal assistance.

Considering the assessment curve of child 1, it can be seen that the child was able to carry out the first three sets successfully without any external support, but required occasional minimal support during the last two sets. This is due to the fact that the first three sets were carried out in the same order as that of the training, the fourth set in reverse order and the fifth set in random order. This could also be due to the fact that the combinations of the colours were different from the way it was taught, but shows that this helps the child to generalise in everyday life. Child 3 and child 5 showed similar trends in the assessment curves, as they also required occasional minimal assistance throughout the session. In case of children 2, 4 and 6, it can be seen that the children needed very minimal help during the first three sets of the trials but executed the reverse and random order sets without any kind of assistance, by scoring a consistent 4 which is the top score. As mentioned before, child 7 was diagnosed with mental retardation in addition to autism, hence this child’s performance was not as good as the others. The training session ended with child 7 scoring a constant 1.3 almost throughout the assessment curve. This is because the child required physical assistance for both colour identification and connection of the Velcros and touch assistance for pincer grasp until the end. It can be observed from Figs. 4 and 5 that the score of child 7 has dropped from 2.8 to 1.3, this is because the child required more assistance to perform the tasks in combination of the colours when compared to the individual colours.

After analysing the graphs of all the children, it can be inferred that at the end of the training there was a distinct enhancement in the identification of the four colours, use of pincer grasp and connecting the closures of the shirt correctly.

### Comparison of pre-assessment and post-assessment scores

To study the improvement in the above skills and to check if the children were able to generalise the tasks taught, post-assessment was carried out.

**Fig. 6:**
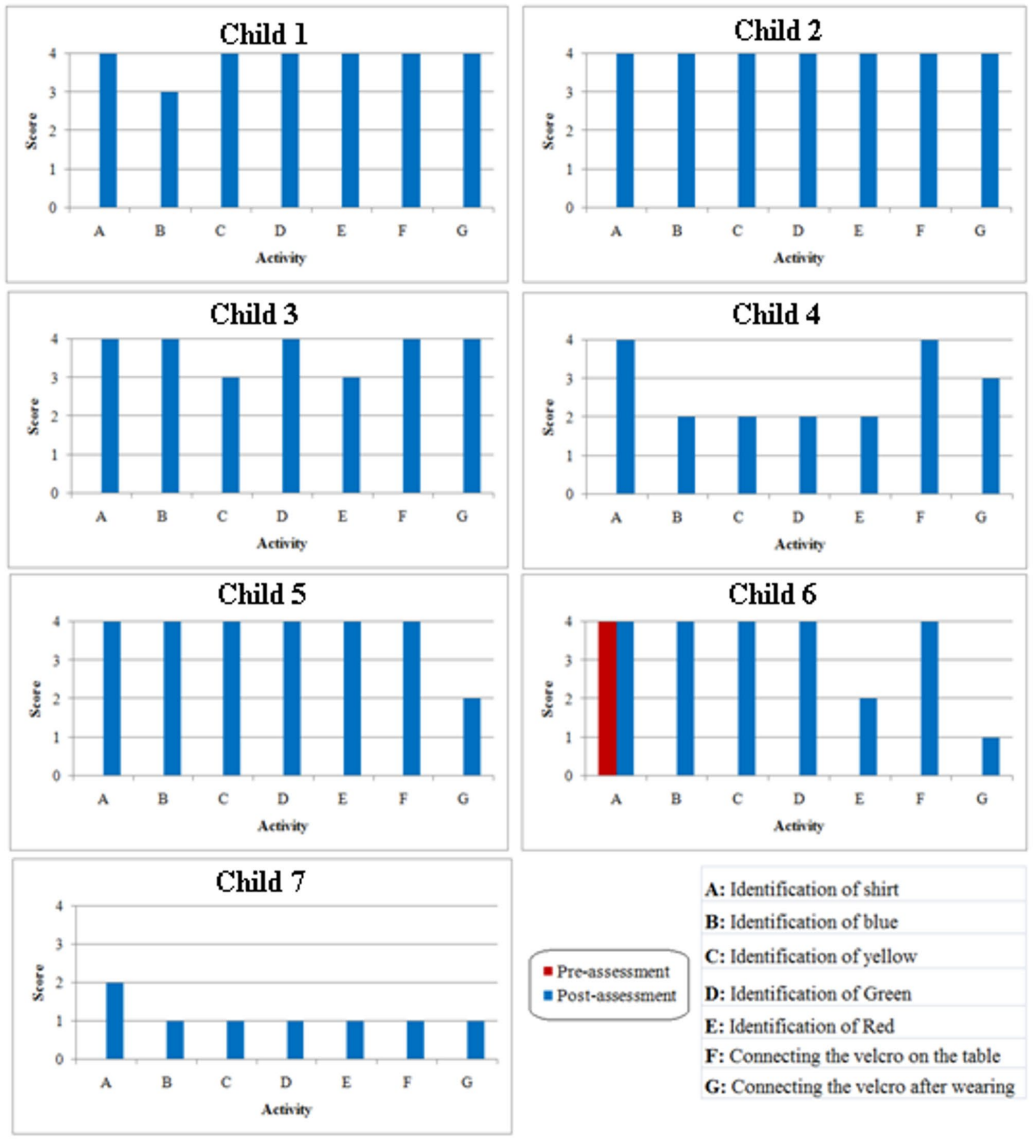
Pre-assessment and post–assessment comparison graph.

Based on the performance and the assistance required, scores were given during the pre-assessment and the post-assessment activities and these outcomes were used to plot the graph as shown in Fig. 6. It can be seen that except for child 6 there was absolutely no performance in the pre-assessment tasks for any of the children. In the post-assessment, except for child 7, every other child had a minimum score of 3 in all the activities. Child 2 executed all the tasks successfully without any assistance. In the case of child 1 and child 3, the former required only minimal assistance (oral cues) to identify the colour blue and the latter required the same to identify yellow and red, but were able to perform the rest without any assistance. Child 5 was able to carry out all the activities successfully except for ‘connecting the Velcro after wearing’ for which the child required touch assistance. In addition to physical assistance for ‘connecting the Velcro after wearing’, child 6 required touch assistance to identify the colour red. Taking child 4 into consideration, it can be seen that the child was able to identify all four colours with touch assistance and required oral cues to connect the Velcros while wearing it. Child 7 did not have any performance in the pre-assessment but, consistent with her training outcome, needed physical assistance to carry out all the activities in the post-assessment. This again attributes to the fact that the child is diagnosed with mental retardation in addition to autism.

**Fig. 7:**
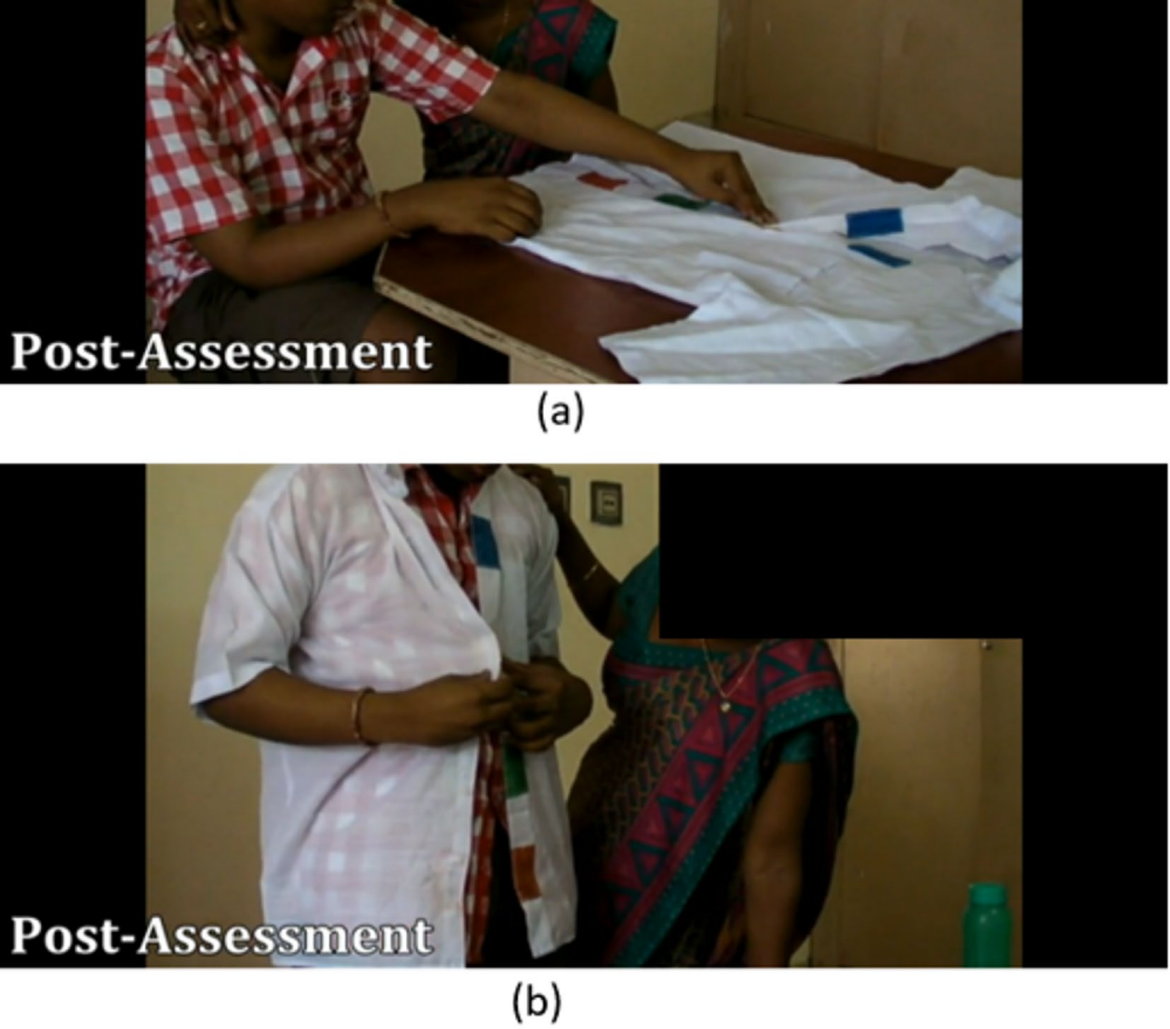
Snapshots from the videos of post-assessment. The child connecting the velcros on the shirt (**a**) on a table and (**b**) worn on him.

In accordance with the explanation in 3.1 Pre-assessment, Fig. 7 shows that child 2 was able to connect the velcros on the shirt, while on the table and once after it was worn on him/her with the help of the trainer. This proves that this device helps in generalising the task even when the orientation of the shirt is altered and this is substantiated by the graphs shown in Fig. 6 of Child 2.

## Conclusion

Autistic children have difficulties in performing daily life activities that demand complex body and brain coordination. Even though there are robotic therapies to impart social skills, only a few studies are being reported that make use of robotic or mechatronic kits for imparting DLS. The mechatronic-shirt kit developed in this work focuses on training an autistic child to identify a shirt and connect the respective velcros once it is worn onto the child by a trainer. Various psychomotor skills addressed here include identification of basic four colours, pincer grasp, elbow movement and hand-eye coordination. The results of the pilot trials conducted were encouraging, and the children were able to generalise the tasks carried out during the trials in daily life. This is affirmed in the post-assessment session as the children were able to identify the real-time shirt and were able to connect the Velcros appropriately after being worn. The results of the post assessment indicate that the children have acquired the identified skills that were previously absent during the pre assessment, reflecting an improvement of around 80%.

## Data Availability

The datasets generated and/or analysed during the current study are not publicly available due to the sensitive nature of the data, which involves personal information of children with disabilities but are available from the corresponding author on reasonable request.
